# Male Sexual Behavior and Pheromone Emission Is Enhanced by Exposure to Guava Fruit Volatiles in *Anastrepha fraterculus*

**DOI:** 10.1371/journal.pone.0124250

**Published:** 2015-04-29

**Authors:** Guillermo E. Bachmann, Diego F. Segura, Francisco Devescovi, M. Laura Juárez, M. Josefina Ruiz, M. Teresa Vera, Jorge L. Cladera, Peter E. A. Teal, Patricia C. Fernández

**Affiliations:** 1 Instituto de Genética “E.A. Favret”, Instituto Nacional de Tecnología Agropecuaria (INTA), Hurlingham, Argentina; 2 Consejo Nacional de Investigaciones Científicas y Técnicas (CONICET), Ciudad Autónoma de Buenos Aires, Argentina; 3 Facultad de Agronomía y Zootecnia (FAZ), Universidad Nacional de Tucumán, San Miguel de Tucumán, Argentina; 4 Estación Experimental Agroindustrial Obispo Colombres (EEAOC), San Miguel de Tucumán, Argentina; 5 Center for Medical, Agricultural and Veterinary Entomology (CMAVE), USDA-ARS, Gainesville, FL, United States of America; 6 Estación Experimental Agropecuaria Delta del Paraná, Instituto Nacional de Tecnología Agropecuaria (INTA), Campana, Argentina; Swedish University of Agricultural Sciences, SWEDEN

## Abstract

**Background:**

Plant chemicals can affect reproductive strategies of tephritid fruit flies by influencing sex pheromone communication and increasing male mating competitiveness.

**Objective and Methodology:**

We explored whether exposure of *Anastrepha fraterculus* males to guava fruit volatiles and to a synthetic blend of volatile compounds released by this fruit affects the sexual performance of wild and laboratory flies. By means of bioassays and pheromone collection we investigated the mechanism underlying this phenomenon.

**Results:**

Guava volatile exposure enhanced male mating success and positively affected male calling behavior and pheromone release in laboratory and wild males. Changes in male behavior appear to be particularly important during the initial phase of the sexual activity period, when most of the mating pairs are formed. Exposure of laboratory males to a subset of guava fruit volatiles enhanced mating success, showing that the response to the fruit might be mimicked artificially.

**Conclusions:**

Volatiles of guava seem to influence male mating success through an enhancement of chemical and physical signals related to the communication between sexes. This finding has important implications for the management of this pest species through the Sterile Insect Technique. We discuss the possibility of using artificial blends to improve the sexual competitiveness of sterile males.

## Introduction

Phytochemicals from host plants are well known to affect behavior and physiology for a wide range of insects [1]. In several phytophagous insect species, males are attracted to volatile compounds released by the host plant, increasing the probability of encountering females [2,3]. However, host plants offer benefits other than cues to find mating partners, and insects have developed different strategies to optimize mating and reproductive success making use of plant chemicals. In some cases, insects acquire host plant chemicals and use them as pheromones or precursors; in others, these chemicals synergize or otherwise induce or enhance the emission of sex pheromones or the response from the receiver, as reviewed in [4,5].

Tephritid fruit flies have evolved a wide range of mating systems, and plants play an important role in shaping male sexual behavior and mating success in many ways [6]. Host plants can act as encounter site for opposite sexes in some species, particularly those having a narrow host range. This is the case of the apple maggot fly, *Rhagoletis pomonella* (Walsh) where males rest on host fruit waiting for the females, with which they mate upon arrival [7]. In several Tephritidae species (typically polyphagous), female location is less predictable, and males aggregate and release sex pheromone in groups (termed leks) to attract females [8–10]. Plant compounds have also been found to affect sexual communication in these lekking species. *Bactrocera dorsalis* (Hendel) males are known to ingest methyl eugenol, a natural compound found in certain species of plants, which serves as a precursor in the synthesis of sex pheromone and enhance the attraction of females [11–13]. Likewise *Bactrocera tryoni* (Froggatt) showed an enhancement of mating success, most likely related to changes in pheromone composition when males fed on cuelure and zingerone [14,15]. In *Ceratitis capitata* (Wiedemann), the exposure of males to fruit volatiles increases male mating success [16–20]. Although the mechanisms underlying this phenomenon are less understood than for *B*. *dorsalis*, the increase in mating success seems to be related to an increase in male pheromone calling behavior [17,21] and also to an alteration in male-derived olfactory cues [22]. This phenomenon has recently been explored in the olive fly, *Bactrocera oleae* (Gmelin), a monophagous pheromone releasing species, for which the exposure to α-pinene increases mating performance of males and females [23].

Previous work carried out by our group indicated that wild and laboratory males of the South American fruit fly, *Anastrepha fraterculus* (Wiedemann), exposed to guava fruit (*Psidium guajava* L.), one of the preferred hosts, mated more frequently than males that have not been exposed to guava [24]. Additionally wild males that were exposed to guava copulated earlier than non-exposed males. This was true even when males were exposed only to fruit volatiles and were not allowed to contact the fruit [24]. This effect was not tested with wild females. Exposure to other host fruits, such as mango (host for *A*. *fraterculus*) did not enhance male sexual performance. Even when the effect of guava fruit on *A*. *fraterculus* males’ mating success has been reported by Vera et al. [24], the mechanism by which males are able to gain an advantage after exposure to this fruit is largely ignored.

The sexual behavior of *A*. *fraterculus* has been analyzed in some Brazilian and Argentinean populations [25–29]. Mating occurs early in the morning, with most copulations taking place during the first 2 or 3 h after dawn, when males aggregate to form leks to attract females [25]. *Anastrepha fraterculus* leks are similar to those described for other *Anastrepha* species [30] and may be operationally defined as aggregations of at least 3 males calling simultaneously in clearly defined areas, usually from adjacent leaves of a single branch [28]. While calling, males often defend a small territory and exhibit a wide repertory of acoustic, visual, and chemical (pheromonal) signals. As in other *Anastrepha* species, pheromone emission is greatest during the puffing of lateral pouches of expandable pleural abdominal cuticle (which is associated to the extrusion of the salivary glands) and the periodic protrusion of anal tissue to form a pouch [31]. This puffing behavior is accompanied by bursts of rapid wing fanning producing sound and directing air over the body surface, which aids pheromone diffusion [31]. The stimuli eliciting lek formation in *A*. *fraterculus* are unknown, but it has been suggested that light level as well as male signalling behavior, mainly through pheromone release, are involved [25]. Females visit the leks and choose a mating partner [9]. The mechanisms by which females choose a mating partner are not fully understood. However, it has been shown that male copulatory success is related to pheromone calling activity, male location within the lek [28], and specific morphological traits such as eye length [27, 28]. This evidence suggests that female mating decisions can be based on integration of multiple stimuli: chemical, visual and probably vibratory signals. And host plant volatiles may play a role in this decision.

The discovery of natural compounds that enhance male mating competitiveness in phytophagous insects could have important economic relevance, especially when the insects are pests. Several tephritid fruit fly pests are controlled by the Sterile Insect Technique (SIT) [32], in which the mating competitiveness of the released sterile males is critical [33]. Consequently, the development of methods to boost male`s sexual performance has been the focus of intense research. In this context, pre-release exposure to guava volatiles may improve the success of *A*. *fraterculus* males in competitions for mating wild females. However, this phenomenon was described only for laboratory females and there is a lack of knowledge about the mechanisms and the identity of the volatile compounds involved. Here, we aimed to: 1) Evaluate whether exposure to guava volatiles enhanced mating competitiveness in laboratory males competing for wild females (Experiment 1A); 2) Evaluate changes in pheromone release and male calling behavior (visual displays during courtship) after guava exposure (Experiment 1B); 3) Evaluate the temporal pattern of calling behavior in exposed and non-exposed males according to mating status (Experiment 2); and 4) Determine whether the enhancement elicited by guava exposure can be mimicked by an artificial blend containing a subset of components present in guava aroma (Experiment 3). Based on previous findings, we hypothesize that specific compounds released by guava fruit trigger an increase in mating success of males which is mediated by changes in male calling behavior. In addition, we hypothesize that exposed males call more intensely that non-exposed males during the entire period of sexual activity for this species (2–3 h after sunrise). Finally, if the response to guava volatiles is due to specific compounds, then there is a subset of volatiles able to mimic the effect of the fruit aroma.

## Materials and Methods

### Insects

*Anastrepha fraterculus* adult flies were obtained either from a laboratory colony or from the wild. Laboratory flies were reared at INTA Castelar from a colony established at the Agricultural Zoology laboratories (Estación Experimental Agroindustrial Obispo Colombres, Tucumán, Argentina). This colony was initiated in 1997 with pupae obtained from infested guavas collected around Tafí Viejo (Tucumán) [34]. Rearing followed standard procedures using an artificial diet based on yeast, wheat germ, sugar, and agar for larvae and a mixture of sugar and hydrolyzed yeast for adults [35, 36]. Wild flies were recovered from infested guavas collected at Horco Molle, Tucumán (26° 49' 0" S / 65° 19' 0" W). Since collected insects did not involve endangered or protected species, no specific permission was required. Fruits were placed in plastic trays on top of a layer of sand to allow pupation. Twice a week, sand was sieved, and pupae were recovered. Both laboratory and wild pupae were placed under controlled environmental conditions (T: 24 ± 2°C, RH: 70 ± 10%, photoperiod 14L: 10D) until adult emergence.

On the first day after emergence flies were sorted by sex and transferred to plastic containers (with the aid of an aspirator), where they were provided food and water. Females were placed in 1 L plastic cylindrical containers (15 cm tall, 12 cm in diameter) in groups of 25 individuals and fed with the standard diet used for rearing, which contains sugar, hydrolyzed yeast (MP Biomedicals, San Francisco, CA, USA), hydrolyzed corn (ARCOR, Tucumán, Argentina) (4:1:1 ratio) and vitamin E (Parafarm, Buenos Aires, Argentina). Males were placed in 21 L plastic containers (37 x 28 x 21 cm) in groups of 100 individuals and fed with sugar and brewer’s yeast (CALSA, Tucumán, Argentina) (3:1 ratio). Flies included in mating tests were sexually mature (>10 d-old for laboratory males; > 14 d-old for wild males; > 14 d-old for laboratory females; > 18 d-old for wild females) [26,34,37,38].

### Experiment 1. Effect of guava exposure on male mating success, calling behavior and pheromone release

#### Exposure to Guava Volatiles

The general procedure followed Vera et al. [24]. Guava fruits were collected from trees occurring in a natural forest in Horco Molle. Prior to exposure, fruits were washed with tap water, cut in halves, and placed in 250 ml cylindrical plastic containers (6 cm tall, 8 cm in diameter), one medium size fruit (ca. 50 g) per container. Fruit exposure started when flies (wild and laboratory males) were sexually immature. Males had no access to the pulp as a cloth mesh was placed over the opening of the container. The minimum distance between the fruit and the mesh was 0.5 cm. The container was placed in the cages described above that housed the males, adult food, and water. The fruit was replaced every other day until the end of the exposure period, which depended on fruit availability and lasted from 2 to 11 days. Even if the time of exposure was not the same among replicates, further analysis confirmed no correlation of time of exposure with mating success (Pearson Product moment correlation analysis r = -0.224, N = 9, P = 0.562). Fruit exposure ended at least 24 h before the mating test. After the fruit exposure period, the cage was transferred to a clean room in which males were kept until they were tested. Control males were maintained in a different room with similar environmental conditions but were not exposed to guavas.

### Experiment 1A. Effect of guava exposure on male mating success

#### Mating Tests

The effect of prior exposure to guava volatiles on male mating success was evaluated by scoring female mating choice in experimental arenas. Each arena consisted of a 1 L plastic cylindrical container (same as described above) with a screen lid that housed three virgin flies: one male exposed to guava, one control male (not exposed to guava), and one female. Male competitors were of the same age and origin (wild or laboratory) and differed only in their exposure to guava volatiles. Females used were always from the wild. Exposed and non-exposed males were labelled by providing them a diet that had been dyed with a food colorant (Fleibor, Tablada, Buenos Aires, Argentina). Randomly assigned colours identified different male treatments. The morning of the mating tests, under semidarkness, males and females were released in the experimental arena, with males released 15 min before females. Once the experiment was set up, the room lights were turned on (8:30 am) at which time an observer started to monitor continuously the occurrence of mating pairs. Whenever a couple was detected, male colour and time at which copulation started were recorded. All experiments were conducted under laboratory conditions (T: 25 ± 1°C and 70 ± 10% RH). Illumination was provided by fluorescent tubes and natural light coming from a window. Given that Argentinean populations of *A*. *fraterculus* present a narrow period of mating activity early in the morning [24,26,39], each mating test lasted 2 h. The experiment was performed along 9 different days, and each day between 30 and 60 mating arenas were observed. In total, we evaluated 459 mating arenas. Sample size is 130 for laboratory males (along 3 different days) and 329 for wild males (along 6 different days) respectively.

### Experiment 1B. Effect of guava exposure on male calling behavior and pheromone release

This experiment examined potential changes in male behavior and physiology related to guava exposure that could account for the increase in mating success demonstrated in Experiment 1A. In order to do that, we collected behavioral and pheromonal data from guava exposed and non-exposed males, both for wild and laboratory males. The experiment was performed along 6 different days after the mating test with males from exp 1A. Each replicate consisted of ten males from each treatment (guava exposed mated males vs. non-exposed unmated ones), placed in a 250 mL glass chamber (20 cm length, 4 cm in diameter). Males were provided with water and sugar solution (10%) until the next day. Wild and laboratory males were placed in separate chambers.

Behavioral recordings and collection of volatiles were performed simultaneously and started at 8:30 am of the day following mating tests and lasted for 3 h [40]. Two components of male courtship associated with pheromone emission and dispersion [31,29] were recorded: wing fanning and exposure of salivary glands. During the 3-h observation period, the number of males performing these behaviors was recorded. Six replicates along six different days were carried out (3 with wild and 3 with laboratory males).

The volatiles emitted by calling males were collected during 3 h from groups of 10 males. A purified air stream was blown over living males enclosed in the glass chambers. Volatiles were collected onto traps made of 30 mg of Hayesept Q adsorbant (Grace, Deerfield, IL, USA). The air flow directed through the apparatus was set at 400 ml/min. After collection, the trapped volatile compounds were eluted with 200 μl of methylene chloride. No internal standard was added. Samples were analyzed chemically by using an Agilent 7890A gas chromatograph (GC) equipped with a HP-5 column (30 m ± 0.32 mm inner diameter ± 0.25 μm film thickness; Agilent Technologies), and a flame ionization detector. The initial oven temperature was 35°C and after 1 min the oven temperature was increased to 100°C at 5°C min^-1^ and from 100°C to 230°C at 12°C min^-1^, then held for 10 min. Samples were injected in the splitless mode with the injector purged at 30 sec with nitrogen as the carrier gas at 27.6 cm/sec flow velocity. To estimate pheromone emission, four compounds characteristic of *A*. *fraterculus* pheromone (anastrephin, epianastrephin, suspensolide, and (*E*,*E*)-α-farnesene), were quantified [41,42].

Compound identities were confirmed by comparison of retention time with those of authentic synthetic samples obtained from the Center for Medical, Agricultural and Veterinary Entomology (USDA-ARS, Gainesville, FL, USA). Seven replicates along 6 different days were carried out. Three with laboratory and four with wild males (2 of the replicates were performed during the same day).

### Experiment 2. Effect of guava exposure on the temporal pattern of wild males calling behavior

Bearing in mind that for *A*. *fraterculus* mating behavior is an activity limited to a specific time during the day, we set up this experiment in order to evaluate if the temporal pattern of male calling behavior is correlated to the temporal pattern of the formation of mating pairs. This experiment was carried out with a new set of wild males. Males were exposed to guava volatiles following the general procedures described above. Exposure was initiated when males were sexually immature (4 d-old) and lasted 4 d. After exposure, males were transferred to new cages and provided with food and water until they were evaluated in a mating test as described above. Non- exposed males were used as control. Immediately after mating tests, females were discarded, and males were assigned to one of the following categories: 1) exposed males that mated (E+), 2) exposed males that did not mate (E-); 3) non-exposed males that mated (noE+); and 4) non-exposed males that did not mate (noE-). To evaluate the temporal pattern of male calling behavior, groups of ten sexually mature 14-d-old males of each treatment (i.e., E+, E-, noE+ and noE-) were placed in 400 mL glass chambers (20 cm length, 4 cm in diameter) the afternoon before the evaluation. A total of 9 chambers (replicates) were prepared for E+ and noE- males and 11 chambers for E- and noE+ males along 5 d. The next morning, after the lights were turned on, the number of males exhibiting fanning, and salivary gland and anal pouch exposure was recorded for males of each treatment. Recordings started at 8.30 h and were carried out every 30 min for a total of 210 min (seven recordings).

### Experiment 3. Effect of an artificial blend exposure on male mating success

#### Blend exposure

The natural aroma from guavas is a complex blend comprising more than 40 compounds [43]. Males were exposed to an artificial blend comprised of a subset of seven synthetic compounds that had been reported in the headspace of guava fruit [43,44]. This experiment involved male exposure to compounds from different chemical families as aldehydes ((*E*)-2-hexenal), esters (ethyl butanoate and ethyl hexanoate), monoterpenes (β-myrcene, limonene, (E)-β-ocimene) and sesquiterpenes (α-humulene). All compounds were obtained from the CMAVE terpene stocks maintained at CMAVE. Samples were either purchased from Sigma-Aldrich (E-2-hexenal, limonene, β-myrcene, (E)-β-ocimene, α-humulene) or synthesized in house (ethyl butanoate, ethyl hexanoate) and purified to a minimum of 98% purity using normal phase HPLC as indicated by analysis by GC-FID and GC-MS. We were able to confirm the presence of all the compounds used in the artificial blend but β-myrcene, in our own samples of guava volatiles (S1 Fig). The blend was prepared by diluting even proportions of the compounds in acetone (99.5% pure). The final concentration for each compound was 10.0 mg/ml [17,45]. A fixed volume (0.1 ml) of the blend was pipetted on a piece of filter paper (Macherey-Nagel, Düren, Germany). Once the acetone had completely evaporated, the filter paper was placed in a 10 mL cylindrical plastic container (3 cm tall, 2 cm in diameter). A thin layer of Parafilm M (Pechiney Plastic Packaging, Chicago, IL, USA), that had been pierced 10 times with an entomological needle, was used to cover the container. This procedure aimed at generating a slow release of the blend. Groups of ten 4-d-old males were exposed to the blend in a 1 L cylindrical plastic container (same as in Experiment 1) with adult food and water for 4 d. The odour source was replaced every other day. Thus, during those 4 d each group of 10 males was exposed to a total of 2.0 mg of each compound. Control males were exposed to acetone only, following the same procedure than exposed males. Mating success was evaluated as described in Experiment 1 except that laboratory females were tested with laboratory males and wild females were tested with wild males. It is noticeable that wild females were used along this work whenever possible. However, guava plant fruiting period is very short in Argentina (1–2 months) and it was not possible to carry out the whole series of experiments with wild females due to availability. Males were marked on the thorax with a dot of water-based paint 48 h (Témpera Alba, Industrias para el Arte S.A, Argentina) before the test to allow for identification [26]. Males that belong to one treatment were painted, whereas males of the other treatment were not. The marked male could be exposed or non-exposed and that was alternated across trials to avoid biases. The experiment lasted 10 days, 3 with laboratory and 7 with wild males. In total, we evaluated 912 mating arenas. Sample size was 330 for laboratory males and 582 for wild males respectively.

### Data analysis

In Experiments 1A and 3, the frequency of mating pairs obtained by males exposed to guava or to the artificial blend and non-exposed males were compared by means of a Yates-corrected G-test of goodness of fit. Tests were carried out separately for laboratory and wild males. In Experiment 1A the distribution of matings along time was compared by means of Chi square test of goodness of fit, for wild and laboratory males separately.

In Experiment 1B, the number of males performing wing fanning and exposing their salivary gland as well as an estimation of the amount of pheromone released were compared between exposed non-mated and non-exposed mated males by means of a mixed effect model. For each replicate the average number of males performing a given behavior across observation bouts was used as the dependent variable while the exposure condition was used as the fixed factor and the origin of the male and the day in which the trial was performed, so as to group cohorts, as the random factors. The amount of pheromone released was estimated as follows: First, the amount of (E,E)-α-farnesene was calculated by performing an external calibration curve using synthetic standards. Afterwards, the amount of suspensolide, anastrephin and epianastrephin was calculated relative to the amount of (E,E)-α-farnesene by obtaining a ratio as follows: area of suspensolide divided by area of farnesene within each chromatogram (same with anastrephin and epianastrephin). Additionally, Pearson Product-Moment correlation analysis was performed in order to study possible correlations among behavioral parameters of male calling and the emission of individual pheromone compounds.

In Experiment 2, the effect of guava exposure on the temporal pattern of male calling behavior along the period of sexual activity was evaluated by means of a General Linear Model (GLM) in which the male exposure condition (i.e., exposed and non-exposed), mating status (i.e. mated or virgin) and the observation period were the fixed factors. The model included all possible interactions and considered the correlation among observations from the same glass chamber. The response variables analyzed were number of males exhibiting fanning, salivary gland exposure, anal pouch exposure and the sum of the three behaviors (from herein referred to as ‘sexual display’). Analysis was performed with the R interface of Infostat [46].

## Results

### Experiment 1A. Effect of guava exposure on male mating success

**Fig 1:**
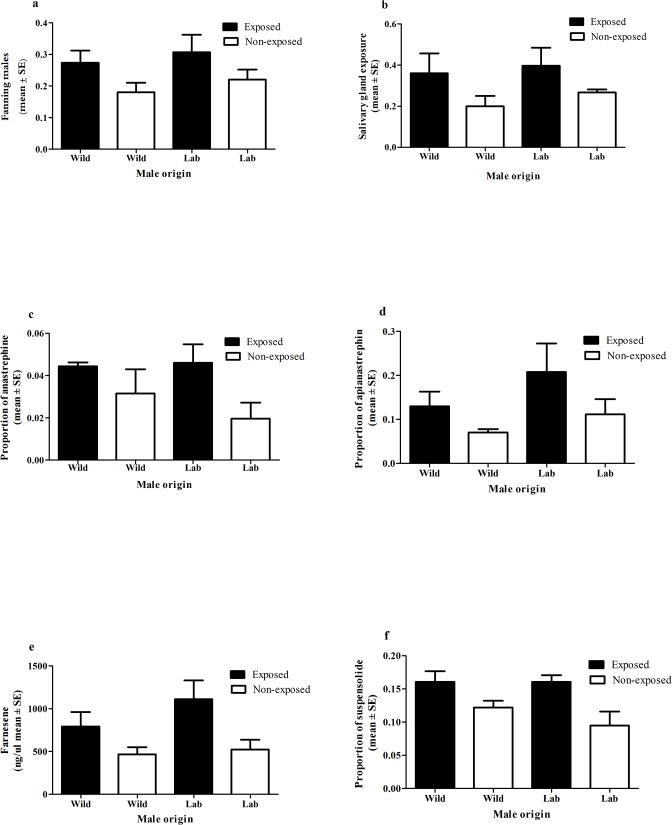
Effect of guava exposure on male mating success and time of mating pair formation. a) Percentage of matings obtained by guava-exposed and non-exposed *Anastrepha fraterculus* males for wild and laboratory populations (asterisks indicate G-Test: P < 0.001, N = 130 for laboratory and N = 329 for wild males). b) Percentage of matings according to time of mating pair formation for guava-exposed and non-exposed males (Chi square Test P < 0.001 for both, laboratory and wild males). In all cases wild females were used in the mating tests.

Exposed males achieved significantly more percentage of mating couples than non-exposed ones irrespective to male origin (laboratory males: Gy = 16.28, N = 130, P<0.001; wild males: Gy = 28.45, N = 329, P<0.001) (Fig 1A). The ratio of mating success between exposed and non-exposed laboratory males was 3 to 1, while for wild males it was 2.3 to 1. This suggests that the enhancing guava effect is slightly stronger in laboratory males. The highest rates of mating pair formation occurred during the first 30 minutes of observation for both, exposed and non-exposed males (Fig 1B) and decreased markedly during the second half of the first hour of observation (30 to 60 minutes interval). After 60 minutes there was almost no mating initiation. Both for laboratory and wild males, highly significant differences were found along time (laboratory males: Chi^2^ = 318.7, N = 130, P<0.001; wild males: Chi^2^ = 99.6, N = 329, P<0.001).

### Experiment 1B. Effect of guava exposure on male calling behavior and pheromone release

**Fig 2:**
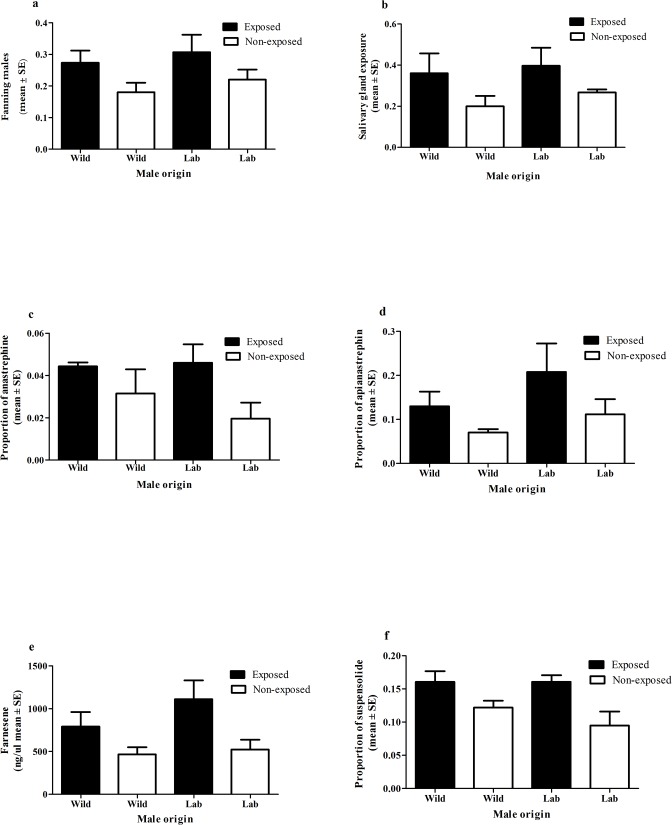
Effect of guava exposure on male calling behavior and pheromone release. a) Number of males that were detected fanning their wings across the observational period (mean ± SE) (mixed effect model P<0.001). b) Number of males that were detected exposing their salivary glands across the observational period (mean ± SE) (mixed effect model P = 0.076). c) Anastrephin released per male (mean proportion to *(E*,*E)*-α-farnesene ± SE) (mixed effect model P = 0.040). d) Epianastrephine released per male (mean proportion to *(E*,*E)*-α-farnesene ± SE) (mixed effect model P = 0.0104). e) Amount (ng) of *(E*,*E)*-α-farnesene released per male SE) (mixed effect model P = 0.083). f) Suspensolide released per male (mean proportion to *(E*,*E)*-α-farnesene ± SE) (mixed effect model P = 0.005). In all cases male origin factor and interaction were non-significant.

The mean number of males performing wing fanning was significantly higher in guava-exposed mated compared to non-exposed unmated males (F_1,5_ = 49, N = 6, P<0.001) (Fig 2A). The mean number of males exposing their salivary glands showed the same tendency, but differences between exposed and non-exposed males were non-significant (F_1,5_ = 5.0, N = 6, P = 0.076) (Fig 2B). The amount of (E,E) -α-farnesene, anastrephin, epianastrephin and suspensolide released was always higher for guava-exposed mated males (Fig 2C–2F). These differences were statistically significant for all compounds, except for (E,E) -α-farnesene (anastrephin: F_1,6_ = 6.80, N = 7, P = 0.040; epianastrephin: F_1,6_ = 13.53, N = 7, P = 0.010; (E,E)-α-farnesene: F_1,6_ = 4.33, N = 7, P = 0.083; suspensolide: F_1,6_ = 9.09, N = 7, P = 0.005). The impact of male origin was in all cases irrelevant (less than 1% of the total variance. Correlations among parameters related to the male calling behavior (i.e. wing fanning and salivary glands exposure) and individual pheromone compounds were all significant with P<0.05 except for wing fanning vs. anastrephin (P = 0.09, S1 Table).

### Experiment 2. Effect of guava exposure on the temporal pattern of wild males calling behavior

**Fig 3:**
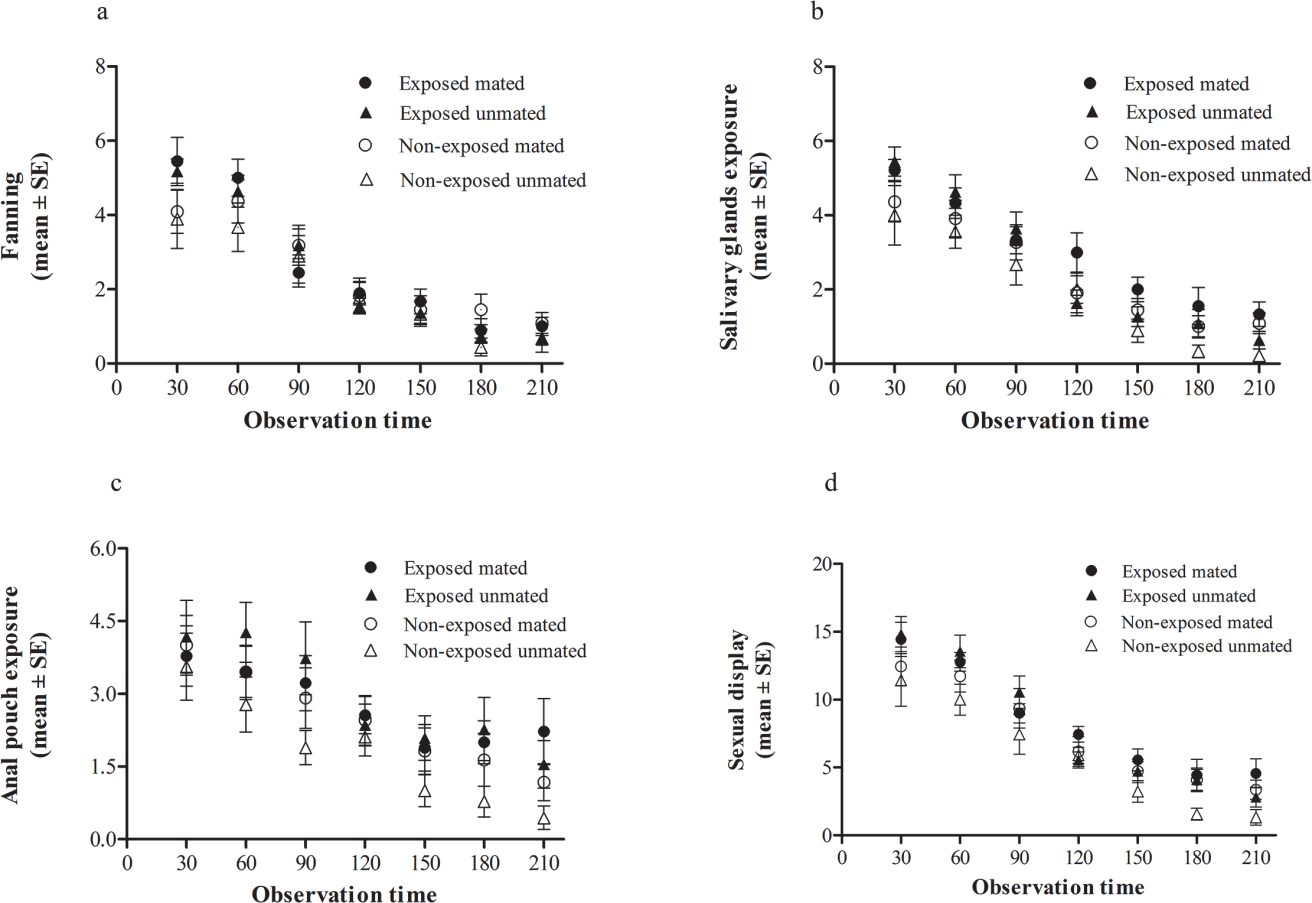
Temporal pattern of the different components of *Anastrepha fraterculus* wild male´s sexual behavior. a) Number of males fanning their wings along the 210 minutes of observation time (mean ± SE) (GLM, P = 0.223 for wing fanning). b) Number of males exposing their salivary gland (mean ± SE) (GLM, P = 0.007 for salivary gland). c) Number of males exposing their anal pouch (mean ± SE) (GLM, P = 0.080 for anal pouch). d) Sexual displays which results from adding the three recorded behavioral parameters (mean ± SE) (GLM, P = 0.024 for sexual display). Mean values are presented separately in all male treatments. In all cases GLM showed significant differences at P<0.0001 for observation time and non-significant differences neither for mating status, nor for any interaction.

Frequency of fanning, salivary gland exposure and anal pouch exposure were high early in the morning and diminished gradually over time (Fig 3). Accordingly, there was a highly significant effect of the observation time on the three recorded behaviors and their sum (i.e. sexual display) (Table 1). The highest rates of sexual display occurred early in the morning, which corresponds to the time of highest mating probability (see Fig 1B). Exposed males showed the highest mean values of the three behaviors analyzed during the first 60 minutes of observation, while the curve for non-exposed and non-mated males showed the lowest values. Salivary gland exposure (Fig 3B) and sexual display (Fig 3C) showed to be significant in guava-exposed males. Mating status showed to be non-significant in any case (Table 1). Given that most of copulations occurred early in the morning we did a two-way ANOVA only for the first observation period (i.e. first 30 minutes from the beginning of sexual activity) as a post hoc test. Results confirmed the occurrence of significant differences between exposed and non-exposed males for salivary gland exposure (F_1,36_ = 4.72, N = 40, P = 0.036) and extended this effect to wing fanning (F_1,36_ = 4.92, N = 40, P = 0.033). For sexual display and anal pouch the effect of guava exposure was not significant (F_1,36_ = 3.22, N = 40, P = 0.081 and F_1,36_ = 0.09, N = 40, P = 0.770 respectively). For the four response variables both, mating status and the interaction between exposure condition and mating status were non-significant (P>0.05).

**Table 1:** General Linear Model analysis for experiment 2.

		Wing fanning		Salivary gland exposure		Anal pouch exposure		Sexual display	
	df	F	P	F	P	F	P	F	P
Exposure condition	1	1.49	0.2232	7.28	0.0074	3.08	0.0803	5.19	0.0236
Mating status	1	1.18	0.2791	3.59	0.0594	0.64	0.4256	1.99	0.1598
Observation period	6	36.61	<0.0001	39.65	<0.0001	8.83	<0.0001	40.27	<0.0001
Exp*MS	1	0.12	0.7274	0.15	0.6971	1.04	0.3097	0.63	0.4270
Exp*Obs	6	1.84	0.0913	0.56	0.7635	1.07	0.382	0.61	0.7239
MS*Obs	6	0.81	0.5639	0.46	0.8381	0.27	0.9493	0.34	0.9139
Exp*MS*Obs	6	0.82	0.5553	1.22	0.2962	0.72	0.6314	1.71	0.1193

The effect of guava exposure on the temporal pattern of male calling behavior of *Anastrepha fraterculus* males was evaluated along the period of sexual activity. The variables considered were wing fanning, salivary and anal pouch exposure, and sexual display (i.e. the sum of the three previously mentioned behaviors). Exp: exposure condition; MS: mating status; Obs: observation period.

### Experiment 3. Effect of an artificial blend exposure on male mating success

**Fig 4:**
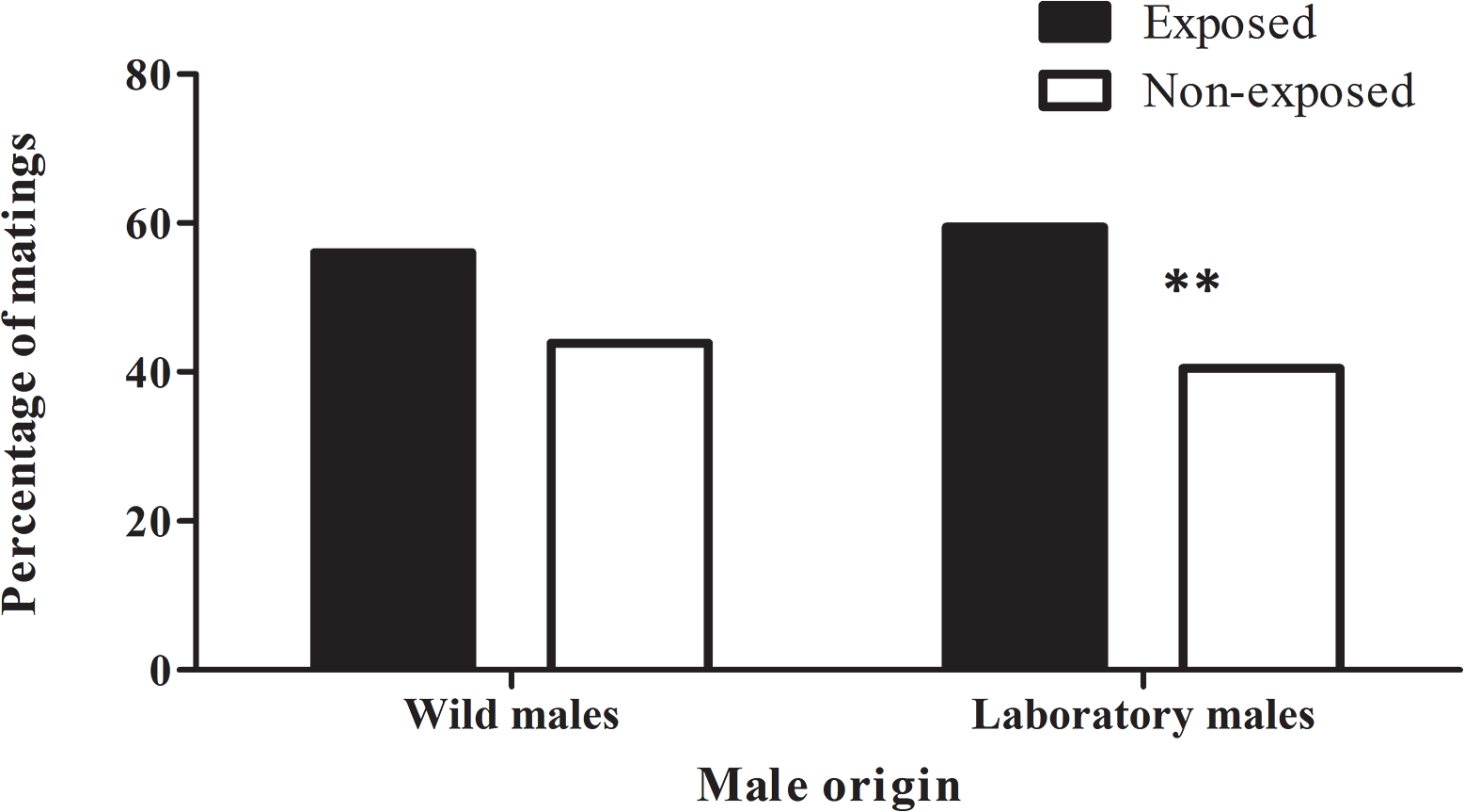
Effect of artificial blend exposure on male mating success. Percentage of matings obtained by wild and laboratory *Anastrepha fraterculus* males, exposed and non-exposed to an artificial blend containing a subset of volatile compounds released by guava fruits ((*E*)-3-hexenal, β-myrcene, (*E*)-β-ocimene, ethyl butanoate, ethyl hexanoate, α-humulene, and limonene). Asterisks indicate G-Test, P = 0.005, N = 330 for laboratory males. For wild males, G-Test, P = 0.179, N = 582. In mating tests, wild females were used with wild males, while laboratory females were used with laboratory males.

The number of matings obtained by laboratory reared males exposed to artificial blend was significantly higher than that obtained by non-exposed males (Gy = 7.905, N = 330, P = 0.005) (Fig 4). These correspond to a 58.7% of mating in exposed lab males vs. 41.3% of mating to non-exposed ones. No differences were detected in the number of matings obtained for exposed and non-exposed wild males (Gy = 1.809, N = 582, P = 0.179) (Fig 4).

## Discussion

The present study confirmed that exposure to guava fruit volatiles increases *A*. *fraterculus* male mating success. Moreover we provided evidence that guava exposure affects behavioral and physiological parameters that are normally correlated with sexual performance. This strongly suggests that guava directly influences male mating competitiveness by altering chemical and physical cues related to the communication between sexes. Changes in male calling behavior appeared to be more marked during the initial phase of the sexual activity period, when most of the mating pairs are formed. The enhanced mating success was partially mimicked in laboratory males by using a subset of volatile chemicals present in guava fruit.

The effect of guava volatiles on *A*. *fraterculus* male mating success was evident both for wild and laboratory flies, being slightly stronger in laboratory flies. Thus, we validated the results of Vera et al. [24] and extended them to laboratory males paired with wild females. In the case of guava volatiles, the mating ratio between exposed and non-exposed males was higher for laboratory than for wild males. In the case of the artificial blend, the response was evident only for laboratory flies. The lower response of wild males could be related to the expected larger variation in the nutritional environment in which they grew as larvae. Wild flies were recovered from guava fruit, a much more variable developing substrate than the artificial larval diet in which laboratory flies were reared. This results in a higher intra-specific difference in male nutritional status at the time of emergence and also in larger variation in male size which has been shown to have an impact on mating success [27]. Alternatively, it can be proposed that wild males are naturally more competitive than laboratory males [47] and the room for improvement after guava exposure is narrower.

We found differences in male calling behavior and pheromone emission that partially unravel the physiological mechanisms responsible for the increased mating success after guava volatiles exposure. Fruit exposure increased both physical (visual and/or mechanical) and chemical (pheromone release) aspects of sexual signalling. Kumaran et al. [14,15] showed that pheromone composition changes after exposure to compounds that sexually enhance males of *B*. *tryoni*, but in their case the males ingested those compounds. In *Toxotrypana curvicauda*, the maximum peak and the temporal pattern of the pheromonal compound 2-methyl-6-vinylpyrazine released were modified by the presence of host fruit and conspecific males or females [48]. Therefore, to our knowledge this is the first demonstration that pheromone emission is increased in males that have been exposed to volatiles of a natural source (with which they did not have contact) that enhance their mating competitiveness in Tephritidae flies. Kumaran et al. [14,49] suggest that complex physiological changes in *B*. *tryoni* occurred when male flies fed on cuelure and zingerone. These changes are not exclusively associated to the pheromone system but also with physiological increase in males activity, suggesting that induced sexual selection is governed not only by female preference but also male competitive mechanisms. Likewise, Shelly [17] and later Papadopoulos et al. [21] observed that males of the Mediterranean fruit fly exposed to ginger root oil (GRO) performed more calling than non-exposed males, but females were equally attracted to pheromone released by GRO-exposed and non-exposed males, suggesting that other stimuli may be needed to trigger female´s mating preference. As found by Kumaran et al. [14] for *B*. *tryoni*, in *A*. *fraterculus* guava exposure affected pheromone emission and the sexual activity of the males through different channels of communication associated with courting (wing fanning, and exposure of salivary gland and anal pouch). However, the fact that pheromone was sampled from a group of males preclude to determine whether guava exposure increases the proportion of males releasing the average amount of pheromone a mature male releases, or it induces individual males to release larger amounts of pheromone beyond an average amount. Yet, collection of pheromone from individual males would help to solve this question. Likewise, data on female behavioral or electrophysiological response to sex pheromone will surely increase our understanding of the mechanisms underlying the effect of guava volatiles on reproduction in *A*. *fraterculus*.

It is important to notice that sexual display and pheromone release were measured one day after the mating test, since it was not possible to measure both mating enhancement and sexual display on the same day given the short mating window (ca. 2 h). Our experiment with wild males (Experiment 2) showed non-significant effect of mating. Additionally, Teal et al. [40] showed that young (6–7 d-old) mated males of *Anastrepha suspensa* (Loew) produce larger amounts of pheromone than virgin males of the same age, but this difference disappears when they are 9 d-old. In our pheromone collection assays tested males were always older than 10 d-old, and thus the differences in pheromone production between exposed and non-exposed males can barely be related to their mating status, reinforcing the idea that higher pheromone emission preceded (and favoured) mating. Hence, we are confident that the increase in pheromone is due to the exposure to guava volatiles.

The expression of sexual display followed a consistent temporal pattern. Activity was high early in the morning, soon after dawn, and diminished with time. Accordingly, mating activity was also greatest within the first 30 minutes of daylight. The effect of guava exposure on wing fanning and salivary gland exposure was especially evident at this time, whereas differences in anal pouch exposure did not became statistically significant along the calling period. These results reveal different regulation of these three behaviors. Nation [31] suggested that the different components of the pheromone are produced and released in different parts of the body in *A*. *suspensa*: anastrephin and epianastrephin are concentrated mainly in the rectal region, while suspensolide and bisabolene were present in the enlarged salivary glands. In *A*. *fraterculus* males, the two isomers of suspensolide and (E,E)*-*α-farnesene were detected from a liquid sample of salivary glands, while anastrephin and epianastrephin were detected only when the insect aeration chamber was washed up with hexane [50]. Together, these results suggest that pheromone emission in *A*. *fraterculus* is a complex task involving multiple points of release and regulation. The fact that there is a correlation among behavioral parameters of male calling and pheromone release suggests a highly coordinated response.

*Anastrepha fraterculus* is an important fruit pest in South America, and the only control method available is the use of pesticides. One environmentally friendly alternative to control this pest is the SIT. Continuous mass-rearing or sterilization may compromise, among others, the capacity to perform elaborate courtship resulting in lower sexual competitiveness [51–54]. Constraints related to mating competitiveness of mass-reared sterilized flies can at least partially be overcome by providing protein supplies, proper radiation doses and irradiation conditions, and methoprene treatment [38,55,56,57]. Pre-release exposure to fruit volatiles may improve the effectiveness of the sterile males as it is currently been conducted for *C*. *capitata* [18, 58]. However, this is a lab study and there were neither competition between wild and mass-reared males, nor sexual enhancement of sterile males. These experiments are the next step of this project since the results will be decisive in order to go further with its implementation.

While the use of actual guava fruit is not feasible considering the desired scale of sterile male production and release protocols, the appropriate approach is to develop a synthetic blend that includes the key compounds present in guava fruit. Similarly to what was found by Kouloussis et al. [44] for *C*. *capitata*, *A*. *fraterculus* laboratory males exposed to an artificial mixture based on compounds present in guava volatiles achieved *ca*. 60% of the recorded mating when they competed with non-exposed males. This result encourage performing additional studies with mass reared irradiated or unfertile males and the identification of the active ingredients in the blend and their ratio in an optimized mixture, to achieve maximum effect in enhancing mating competitiveness to be transferred to a massive scale.

## Supporting Information

S1 Fig Total ion chromatogram obtained from analysis of volatiles collected from guava fruit. The fruit was cut in halves and placed in cylindrical glass chambers of 0.8 L (40 cm long, 5 cm in diameter). Volatile collection procedure was identical to those followed for male pheromone collection. Volatile traps (Hayesept Q) were eluted with 200 μl of methylene chloride and chemically analyzed using an Agilent 6890 instrument coupled to Agilent 5973 selective mass detector. Compounds are (1) Ethyl butanoate, (2) *E*-2-hexenal, (3) Ethyl hexanoate, (4) Limonene, (5) *E*-β-ocimene, (6) α-humulene.
(PDF)

S1 Table Correlation values among behavioral parameters of male calling and individual pheromone compounds. Results of the Pearson Product-Moment correlation analysis. Correlation coefficient *r* and associated *P*-value is presented for each pair of variables. N = 16.
(DOCX)

## References

[1] Schoonhoven LM, van Loon JJA, Dicke M. Insect-Plant Biology. 2nd ed. U.K: Unibooks. Oxford University Press; 2005.

[2] Thornhill R, Alcock J. The evolution of insect mating system Cambridge: Harvard University Press; 1983.

[3] Bernays EA, Chapman RF. Host Plant Selection by Phytophagous Insects. New York: Chapman and Hall, editors; 1994.

[4] Landolt PJ, Phillip TW. Host plant influences on sex pheromone behavior of phytophagous insects. Annu Rev Entomol. 1997; 42: 371–91. 15012318 10.1146/annurev.ento.42.1.371

[5] Reddy GVP, Guerrero A. Interactions of insect pheromones and plant semiochemicals. Trends Plant Sci. 2004; 9: 253–261. 15130551 10.1016/j.tplants.2004.03.009

[6] Diaz-Fleisher F, Aluja M. Behavior of Tephritid flies: A Historical Perspective In: Fruit flies (Tephritidae): phylogeny and evolution of behavior. CRC, Washington, DC 2000; 39–72.

[7] Prokopy RJ, Bennett EW, Bush GL. Mating behavior in *Rhagoletis pomonella* (Diptera: Tephritidae). Can Entomol. 1971; 103: 1405–1409.

[8] Prokopy RJ. Mating behavior of frugivorous Tephritidae in nature. Proc of the International Congress of Entomol Symposium of Fruit Fly Problems. 1980; 16: 37–46.

[9] Burk T. Signalling and sex in acalypterate flies. Fla Entomol. 1981; 66: 30–43.

[10] Shelly TE, Kaneshiro KI. Lek behavior of the oriental fruit fly en Hawaii. J Insect Behav. 1991; 4: 235–241.

[11] Shelly TE, Dewire AM. Chemically mediated success in male Oriental fruit flies (Diptera: Tephritidae). Ann Entomol Soc Am. 1994; 87: 375–382.

[12] Tan KH, Nishida R. Sex pheromone and mating competition after methyl eugenol consumption in the *Bactrocera dorsalis* complex In: Fruit fly pests—a world assessment of their biology and management. St. Lucie Press, Delray, FL USA 1996; 147–153.

[13] Nishida R, Shelly TE, Kaneshiro KY. Acquisition of female attractive fragrance by males of the oriental fruit fly from a Hawaiian lei flower, *Fagraea berteriana* . J Chem Ecol. 1997; 23: 2275–2285.

[14] Kumaran N, Balagawi S, Schutze MK, Clarke AR. Evolution of lure response in tephritid fruit flies: phytochemicals as drivers of sexual selection. Anim Behav. 2013; 85: 781–789.

[15] Kumaran N, Hayes RA, Clarke AR. Cuelure but not zingerone make the sex pheromone of male *Bactrocera tryoni* (Tephritidae: Diptera) more attractive to females. J Ins Physiol. 2014; 10.1016/j.jinsphys.2014.06.015 25010549

[16] Papadopoulos NT, Katsoyannos BL, Kouloussis NA, Hendrichs J. Effect of orange peel substances on mating competitiveness of male *Ceratitis capitata* . Entomol Exp Appl. 2001; 99: 253–261.

[17] Shelly TE. Exposure to alpha-copaene and alpha-copaene-containing oils enhances mating success of male Mediterranean fruit fly (Diptera: Tephritidae). Ann Entomol Soc Am. 2001; 94: 497–502. 10.1603/0022-0493-94.6.1413 11777043

[18] Shelly TE, McInnis DO. Exposure to ginger root oil enhances mating success of irradiated, mass-reared males of Mediterranean fruit fly (Diptera: Tephritidae). J Econ Entomol. 2001; 94: 1413–1418. 11777043 10.1603/0022-0493-94.6.1413

[19] Shelly TE, Villalobos E. Host plant influence on the mating success of male Mediterranean fruit flies: variable effects within and between individual plants. Anim Behav. 2004; 68: 417–426.

[20] Shelly TE, Cowan AN, Edu J, Pahio E. Mating success of male Mediterranean fruit flies following exposure to two sources of α-copaene, manuka oil and mango. Fla Entomol. 2008; 91: 9–15.

[21] Papadopoulos NT, Shelly TE, Niyazi N, Jang E. Olfactory and behavioral mechanisms underlying enhanced mating competitiveness following exposure to ginger root oil and orange oil in males of the Mediterranean fruit fly, *Ceratitis capitata* (Diptera: Tephritidae). J Insect Behav. 2006; 19: 403–418.

[22] Shelly TE, Edu J, Pahio E, Nishimoto J. Scented males and choosy females: does male odor influence female mate choice in the Mediterranean fruit fly? J Chem Ecol. 2007; 33: 2308–2324. 18030532 10.1007/s10886-007-9394-y

[23] Gerofotis CD, Ioannou CS, Papadopoulos NT. Aromatized to Find Mates: a-Pinene Aroma Boosts the Mating Success of Adult Olive Fruit Flies. PLoS ONE. 2013; 8(11): e81336 10.1371/journal.pone.0081336 24260571 PMC3834339

[24] Vera MT, Ruiz MJ, Oviedo A, Abraham S, Mendoza M, Segura DF, et al Fruit compounds affect male sexual competitiveness in the South American fruit fly, *Anastrepha fraterculus* (Diptera: Tephritidae). J Appl Entomol. 2013; 137(1): 2–10.

[25] Malavasi A, Morgante JS, Prokopy RJ. Distribution and activities of *Anastrepha fraterculus* (Diptera: Tephritidae) flies on host and non host trees. Ann Entomol Soc Am. 1983; 76: 286–292.

[26] Petit-Marty N, Vera MT, Calcagno G, Cladera JL, Segura DF, Allinghi A, et al Sexual behavior and mating compatibility among four populations of *Anastrepha fraterculus* (Diptera: Tephritidae) from Argentina. Ann Entomol Soc Am. 2004; 97: 1320–1327.

[27] Sciurano RB, Segura DF, Rodriguero M, Gómez Cendra P, Allinghi A, Cladera JL, et al Sexual selection on multivariate phenotype in *Anastrepha fraterculus* (Diptera: Tephritidae) from Argentina. Fla Entomol. 2007; 90: 163–170.

[28] Segura DF, Petit-Marty N, Sciurano RB, Vera MT, Calcagno G, Allinghi A, et al Lekking behavior of *Anastrepha fraterculus* (Diptera: Tephritidae). Fla Entomol. 2007; 90: 154–162.

[29] Gómez Cendra P, Calcagno G, Belluscio L, Vilardi JC. Male courtship behavior of the South American fruit fly, *Anastrepha fraterculus*, from an Argentinean laboratory strain. J Insect Sci. 2011; 11: 175 10.1673/031.011.17501 22958000 PMC3469206

[30] Aluja M, Birke AB. Habitat use by *Anastrepha obliqua* flies (Diptera: Tephritidae) in a mixed mango and tropical plum orchard. Ann Entomol Soc Am. 1993; 18: 799–812.

[31] Nation JL. The role of pheromones in the mating system of *Anastrepha* fruit flies In: World crop pests: fruit flies, their biology, natural enemies and control. Elsevier, Amsterdam 1989; 189–205.

[32] Enkerlin WR. Impact of fruit fly control programmes using the sterile insect technique In: Sterile Insect Technique: Principles and practice in area-wide integrated pest management. IAEA. Springer The Netherlands 2005; 651–676.

[33] Prokopy RJ, Hendrichs J. Mating behavior of *Ceratitis capitata* on a field-caged host tree. Ann Entomol Soc Am. 1979; 72: 642–648.

[34] Jaldo HE. Estudios biológicos y poblacionales de *Anastrepha fraterculus* (Wiedemann) (Diptera: Tephritidae). PhD Thesis, Facultad de Agronomía y Zootecnia, Universidad Nacional de Tucumán, Argentina. 2001.

[35] Jaldo HE, Gramajo C, Willink E. Mass rearing of *Anastrepha fraterculus* (Diptera: Tephritidae): a preliminary strategy. Fla Entomol. 2001; 84: 716–718.

[36] Vera MT, Abraham S, Oviedo A, Willink E. Demographic and quality control parameters of *Anastrepha fraterculus* (Diptera: Tephritidae) artificial rearing. Fla Entomol. 2007; 90: 53–57.

[37] Jaldo HE, Willink E, Liedo P. Demographic analysis of mass-reared *Anastrepha fraterculus* (Diptera: Tephritidae) in Tucumán, Argentina. Rev Ind Agríc Tucumán. 2007; 84: 15–20.

[38] Segura DF, Utgés ME, Liendo MC, Rodríguez MC, Devescovi F, Vera M, et al Methoprene treatment reduces the pre-copulatory period in *Anastrepha fraterculus* (Diptera: Tephritidae) sterile males. J Appl Entomol. 2013; 137(1): 19–29.

[39] Vera MT, Cáceres C, Wornoayporn V, Islam A, Robinson AS, de la Vega MH, et al Mating incompatibility among populations of the South American fruit fly *Anastrepha fraterculus* (Wied.) (Diptera: Tephritidae). Ann Entomol Soc Am. 2006; 99: 387–397.

[40] Teal PEA, Gómez-Simuta Y, Proveaux AT. Mating experience and juvenile hormone enhance sexual signalling and mating in male Caribbean fruit flies. PNAS. 2000; 97: 3708–3712. 10706642 10.1073/pnas.060034397 PMC16304

[41] Cáceres C, Segura DF, Vera MT, Wornoapyorn V, Cladera JL, Teal P, et al Incipient speciation revealed in *Anastrepha fraterculus* (Diptera: Tephritidae) by studies on mating compatibility, sex pheromones, hybridization, and cytology. Biol J Linn Soc. 2009; 97: 152–165.

[42] Břízová R, Mendonça AL, Vanícková L, Mendonça AL, Da Silva CE, Tomčala A, et al Pheromone analyses of the *Anastrepha fraterculus* (Diptera: Tephritidae) cryptic species complex. Fla Entomol. 2013; 96: 1107–1115.

[43] Pino JA, Ortega A, Rosado A. Volatile constituents of guava fruits from Cuba. J Essent Oil Res. 1999; 11(5) 623–629.

[44] Mahattanatawee K, Goodner KL, Baldwin EA. Volatile constituents and character impact compounds of selected florida’s tropical fruit. Proc Fla State Hort Soc. 2005; 118: 414–418.

[45] Kouloussis NA, Katsoyannos BI, Papadopoulos NT, Ioannou CS, Iliadis IV. Enhanced mating competitiveness of *Ceratitis capitata* males following exposure to citrus compounds. J Appl Entomol. 2013; 137 (1): 30–38.

[46] Balzarini MG, Gonzalez L, Tablada M, Casanoves F, Di Rienzo JA, Robledo CW. Manual del Usuario. Córdoba, Argentina: Editorial Brujas. 2008; 336 pp.

[47] Bosa CF, Cruz-López L, Zepeda-Cisneros CS, Valle-Mora J, Guillén-Navarro K, Liedo P. Sexual behavior and male volatile compounds in wild and mass-reared strains of the Mexican fruit fly Anastrepha ludens (Loew) (Diptera: Tephritidae) held under different colony management regimes. Insect Sci. 2014; 10.1111/1744-7917.12180 25339372

[48] Robledo N, Arzuffi R. Influence of host fruit and conspecifics on the release of the sex pheromone by *Toxotrypana curvicauda* males (Diptera: Tephritidae). Environ Entomol. 2012; 41(2): 387–91. 10.1603/EN11037 22507013

[49] Kumaran N, Prentis PJ, Mangalam KP, Schutze MK, Clarke AR. Sexual selection in true fruit flies (Diptera: Tephritidae): transcriptome and experimental evidences for phytochemicals increasing male competitive ability. Molec Ecol. 2014; 10.1111/mec.12880 25112896

[50] de Lima IS, Howse PE, do Nascimento RR. Volatile Substances from Male *Anastrepha fraterculus* Wied. (Diptera: Tephritidae): Identification and Behavioral Activity. J. Braz. Chem. Soc. 2001; 12 (2): 196–201.

[51] McInnis DO, Lance DR, Jackson CG. Behavioral resistance to the sterile insect technique by mediterranean fruit fly (diptera: tephritidae) in Hawaii. Ann Entomol Soc Am. 1996; 89: 739–744.

[52] Cayol JP. Changes in sexual behavior and life history traits of tephritid species caused by mass-rearing processes In: Aluja M, Norrbom AL, editors. Fruit flies (Tephritidae): phylogeny and evolution of behavior. Boca Raton, FL: CRC Press; 2000 pp. 843–860.

[53] Lux SA, Vilardi JC, Liedo P, Gaggl K, Calcagno GE, Munyiri FN, et al Effects of irradiation on the courtship behavior of medfly (Diptera: Tephritidae) mass reared for the sterile insect technique. Fla Entomol. 2002; 85: 102–112.

[54] Rull J, Brunel O, Méndez ME. Mass rearing history negatively affects mating success of male Anastrepha ludens (Diptera: Tephritidae) reared for sterile insect technique programs. J Econ Entomol. 2005; 98: 1510–1516. 16334318 10.1093/jee/98.5.1510

[55] Allinghi A, Calcagno G, Petit-Marty N, Gómez Cendra P, Segura D, Vera T, et al Compatibility and competitiveness of a laboratory strain of *Anastrepha fraterculus* (Diptera: Tephritidae) after irradiation treatment. Fla Entomol. 2007; 90(1): 27–32.

[56] Liendo MC, Devescovi F, Bachmann GE, Utgés ME, Abraham S, Vera MT, et al Precocious sexual signalling and mating in *Anastrepha fraterculus* (Diptera: Tephritidae) sterile males achieved through juvenile hormone treatment and protein supplements. Bull Entomol Res. 2013; 103(1): 1–13. 10.1017/S0007485312000442 22929968

[57] Segura DF, Cáceres C, Vera MT, Wornoayporn V, Islam A, Teal PEA, et al Enhancing mating performance after juvenile hormone treatment in *Anastrepha fraterculus*: a differential response in males and females acts as a physiological sexing system. Entomol Exp Appl. 2009; 131(1): 75–84.

[58] Shelly TE, Edu J, Smith E, Hoffman K, War M, Santos R, et al Aromatherapy on a large scale: exposing entire holding rooms to ginger root oil increases the mating competitiveness of sterile males of the Mediterranean fruit fly. Entomol Exp Appl. 2007; 123: 193–201.

