# Correction: Male Sexual Behavior and Pheromone Emission Is Enhanced by Exposure to Guava Fruit Volatiles in *Anastrepha fraterculus*

**DOI:** 10.1371/journal.pone.0129523

**Published:** 2015-05-29

**Authors:** Guillermo E. Bachmann, Diego F. Segura, Francisco Devescovi, M. Laura Juárez, M. Josefina Ruiz, M. Teresa Vera, Jorge L. Cladera, Peter E. A. Teal, Patricia C. Fernández

Fig 1 is incorrect. Please view the correct Fig 1 here.

**Fig 1:**
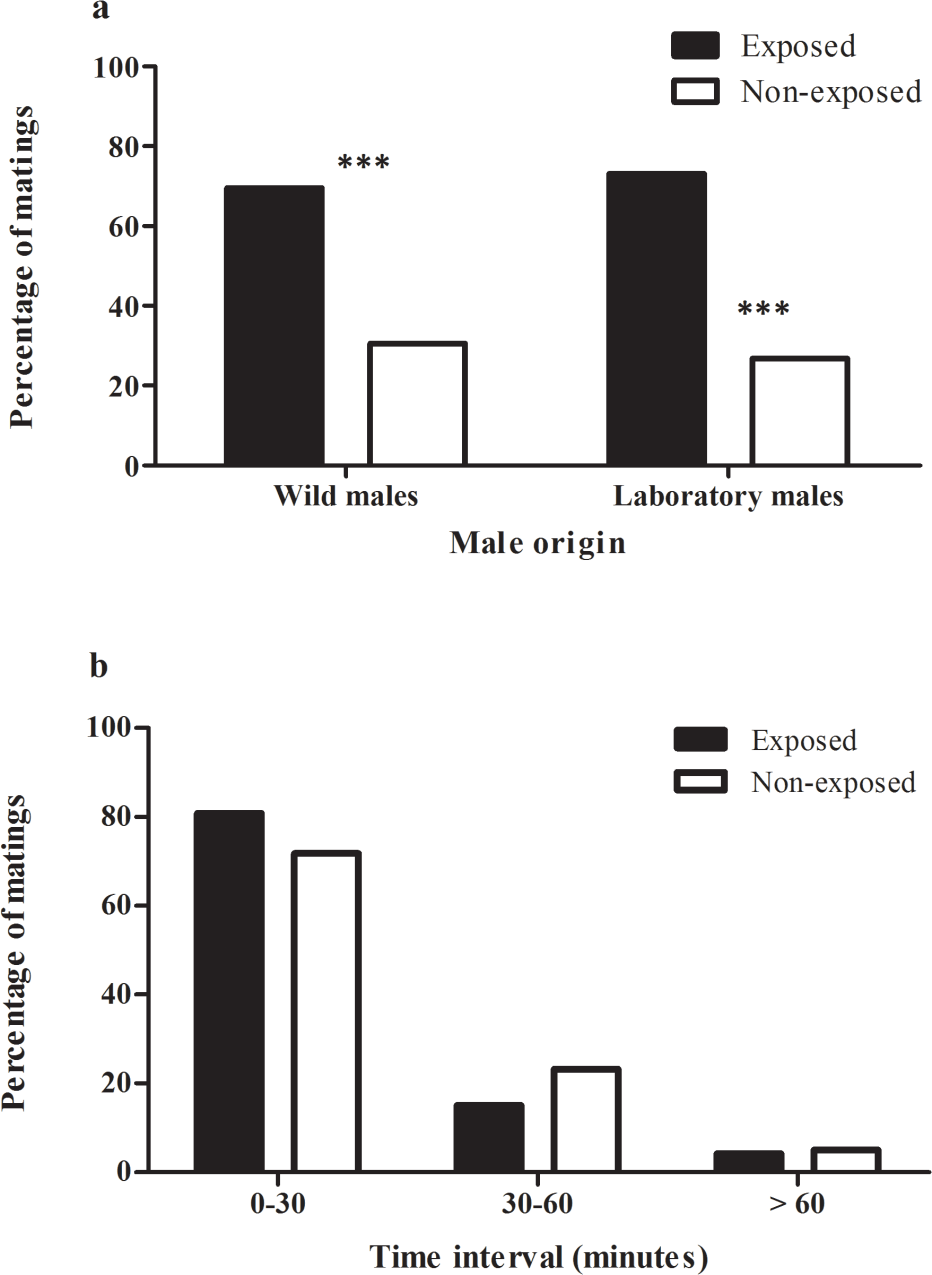
Effect of guava exposure on male mating success and time of mating pair formation. a) Percentage of matings obtained by guava-exposed and non-exposed *Anastrepha fraterculus* males for wild and laboratory populations (asterisks indicate G-Test: P < 0.001, N = 130 for laboratory and N = 329 for wild males). b) Percentage of matings according to time of mating pair formation for guava-exposed and non-exposed males (Chi square Test P < 0.001 for both, laboratory and wild males). In all cases wild females were used in the mating tests.
