# Supplementary figures and images for: Male Sexual Behavior and Pheromone Emission Is Enhanced by Exposure to Guava Fruit Volatiles in Anastrepha fraterculus
Source: PLoS One. 2015 Apr 29;10(4):e0124250. doi: 10.1371/journal.pone.0124250 (PMC4414461; doi:10.1371/journal.pone.0124250)

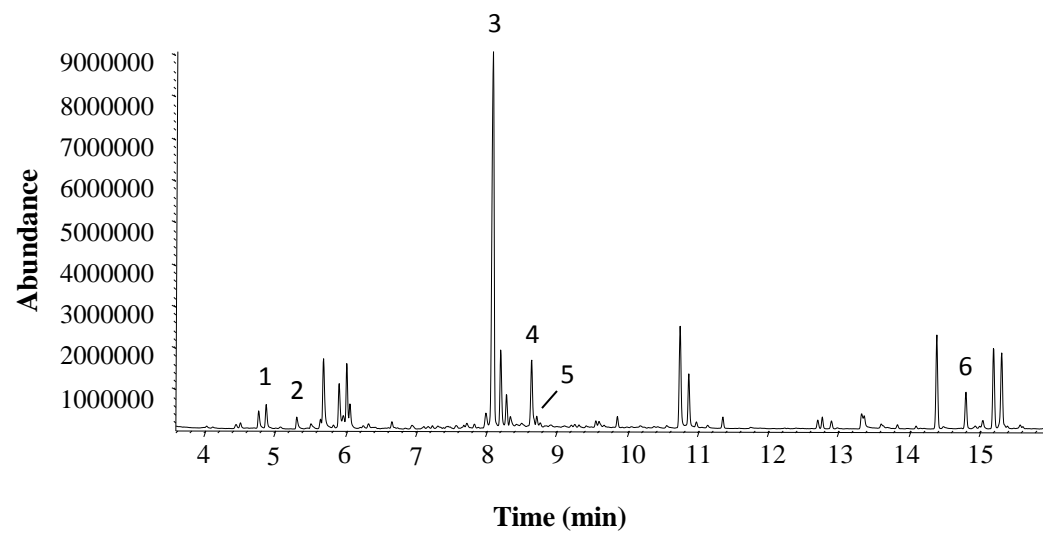

Supplement: S1 Fig — The fruit was cut in halves and placed in cylindrical glass chambers of 0.8 L (40 cm long, 5 cm in diameter). Volatile collection procedure was identical to those followed for male pheromone collection. Volatile traps (Hayesept Q) were eluted with 200 μl of methylene chloride and chemically analyzed using an Agilent 6890 instrument coupled to Agilent 5973 selective mass detector. Compounds are (1) Ethyl butanoate, (2) E-2-hexenal, (3) Ethyl hexanoate, (4) Limonene, (5) E-β-ocimene, (6) α-humulene. (PDF) [file pone.0124250.s001.pdf]
