# Supplementary material for: Male Sexual Behavior and Pheromone Emission Is Enhanced by Exposure to Guava Fruit Volatiles in Anastrepha fraterculus
Source: PLoS One. 2015 Apr 29;10(4):e0124250. doi: 10.1371/journal.pone.0124250 (PMC4414461; doi:10.1371/journal.pone.0124250)
Supplement: S1 Table — Results of the Pearson Product-Moment correlation analysis. Correlation coefficient r and associated P-value is presented for each pair of variables. N = 16. (DOCX) [file pone.0124250.s002.docx]

|  | Anastrephin | Epianastrephin | Suspensolide | (E,E)-α-Farnesene |
| --- | --- | --- | --- | --- |
| Gland exposure | *r*=0.57, P=0.02 | *r*=0.78, P<0.001 | *r*=0.72, P=0.002 | *r*=0.72, P=0.002 |
| Wing fanning | *r*=0.44, P=0.09 | *r*=0.75, P<0.001 | *r*=0.62, P=0.01 | *r*=0.60, P=0.02 |
